# Non-coronary atherosclerosis: a marker of poor prognosis in patients undergoing coronary artery bypass surgery

**DOI:** 10.3389/fcvm.2024.1305162

**Published:** 2024-02-23

**Authors:** Óscar González-Lorenzo, Juan A. Franco Pelaez, Andrea Kallmeyer, Luis Nieto, Laura Esteban, Ana Pello, Álvaro Aceña, Gonzalo Aldamiz, José Tuñón

**Affiliations:** ^1^ Department of Cardiology, IIS-Fundación Jiménez Díaz, Madrid, Spain; ^2^ Faculty of Medicine, Autónoma University, Madrid, Spain; ^3^ Department of Cardiac Surgery, IIS-Fundación Jiménez Díaz, Madrid, Spain; ^4^ Laboratory Department of Vascular Pathology, IIS-Fundación Jiménez Díaz, Madrid, Spain; ^5^ CIBERCV, ISCIII, Madrid, Spain

**Keywords:** peripheral artery disease (PAD), stroke, transient ischaemic attack (TIA), coronary artery bypass grafting (CABG), cardiovascular risk factors

## Abstract

**Introduction:**

The presence of non-coronary atherosclerosis (NCA) in patients with coronary artery disease is associated with a poor prognosis. We have studied whether NCA is also a predictor of poorer outcomes in patients undergoing coronary artery bypass grafting (CABG).

**Materials and methods:**

This is an observational study involving 567 consecutive patients who underwent CABG. Variables and prognosis were analysed based on the presence or absence of NCA, defined as previous stroke, transient ischaemic attack (TIA), or peripheral artery disease (PAD) [lower extremity artery disease (LEAD), carotid disease, previous lower limb vascular surgery, or abdominal aortic aneurysm (AAA)]. The primary outcome was a combination of TIA/stroke, acute myocardial infarction, new revascularization procedure, or death. The secondary outcome added the need for LEAD revascularization or AAA surgery.

**Results:**

One-hundred thirty-eight patients (24%) had NCA. Among them, traditional cardiovascular risk factors and older age were more frequently present. At multivariate analysis, NCA [hazard ratio (HR) = 1.84, 95% confidence interval (CI) 1.27–2.69], age (HR = 1.35, 95% CI 1.09–1.67, *p* = 0.004), and diabetes mellitus (HR = 1.50, 95% CI 1.05–2.15, *p* = 0.025), were positively associated with the development of the primary outcome, while estimated glomerular filtration rate (HR = 0.86, 95% CI 0.80–0.93, *p* = 0.001) and use of left internal mammary artery (HR = 0.36, 95% CI 0.15–0.82, *p* = 0.035), were inversely associated with this outcome. NCA was also an independent predictor of the secondary outcome. Mortality was also higher in NCA patients (27.5% vs. 9%, *p* < 0.001).

**Conclusions:**

Among patients undergoing CABG, the presence of NCA doubled the risk of developing cardiovascular events, and it was associated with higher mortality.

## Introduction

High blood pressure, dyslipidaemia, smoking, diabetes, obesity, and a sedentary lifestyle are well-known risk factors for the development of atherosclerosis, and their prevalence is also related to aging. The association between the burden of these risk factors and the vascular territory affected by atherosclerosis is still under debate. However, there is consensus that once atherosclerosis is established in one vascular territory, the risk of ischaemic events increases in all vascular beds, leading to excess mortality (1).

It has been demonstrated that multiple artery disease results in a worse prognosis (2–4). However, to date, only one clinical trial (5) has been designed to systematically assess the existence of poly-vascular disease among coronary patients, with no clinical benefit of this approach demonstrated.

Notwithstanding this fact, it is beyond doubt that the coexistence of non-coronary atherosclerosis (NCA) worsens the prognosis of CAD patients, even when the first event is not clinically overt. The presence of lower extremity artery disease (LEAD) is known to be an independent risk factor since, after adjusting the analysis for smoking habit, age, diabetes, and gender, it still multiplies by four the risk of death (6, 7). This association appears less clear in patients with carotid artery disease (8).

On the other hand, patients with established CAD are at high risk for cardiac and cerebral ischaemic events, such as acute myocardial infarction (AMI), stroke/transient ischaemic attack (TIA), or even death (9). Furthermore, the presence of CAD is associated with an increased risk of subclinical abdominal aortic aneurysms (AAAs) and future AAA-related adverse events (10, 11). The existence of NCA is known to be associated with poor outcomes in patients with CAD, as it indicates the presence of extensive atherosclerosis (12). The aim of the present study is to analyse whether the presence of NCA among coronary patients undergoing coronary artery bypass graft (CABG) is associated with a poor outcome.

## Materials and methods

### Study design

The design was a retrospective, observational, single-centre study. The study was carried out in a tertiary-level university hospital. Patients who underwent CABG between 2010 and 2018 were included on a consecutive basis, excluding those with concomitant valve or aortic surgery.

Clinical data were reviewed to obtain a wide range of variables concerning demographics, clinical characteristics, blood tests, and surgery-related data. After this, the cohort was divided into two groups, depending on the presence or absence of NCA, defined as previous stroke, TIA, or PAD [lower extremity artery disease (LEAD), carotid disease, previous lower limb vascular surgery, or AAA].

This study conforms to the ethical guidelines of the 1975 Declaration of Helsinki as reflected in *a priori* approval by the human research committee of Fundación Jiménez Díaz Hospital Ethics Board for Research (register number: EO077-19_FJD).

### Events

Follow-up was conducted by a review of the clinical history, during which adverse events were registered. We considered the date of surgery as the beginning of the follow-up. The primary outcome included the combination of TIA/stroke, AMI, new revascularization procedure, or death. The secondary outcome added the need for lower extremity revascularization or AAA surgery to the primary outcome.

### Statistical analysis

Quantitative data are presented as medians and interquartile ranges. Comparisons between groups were performed by Student's *t*-test for variables that met the assumption of normality and by a non-parametric test (Mann–Whitney *U*-test) for those that did not. Normality was assessed by the Kolmogorov–Smirnov test, where *p* < 0.05 indicated that the variable did not meet the assumption of normality. Qualitative variables are displayed as percentages, and groups were compared using the chi-squared test (or Fisher's exact test when appropriate).

To find prognostic factors of primary and secondary endpoints, all relevant clinical and analytical variables, including NCA, were tested in a univariate Cox proportional hazards regression analysis. After that, all variables with a significance level of *p* < 0.2 in univariate analysis were included in a multivariate model. This multivariate analysis was performed using the backward step method, keeping only those variables with a significance level of *p* < 0.05 as calculated by the likelihood ratio method. The effect of every independent predictor variable is presented as a hazard ratio (HR) and its 95% confidence interval (CI). Kaplan–Meier curves were plotted to compare the intervals of survival free of the primary and secondary outcomes in patients with or without NCA at baseline, and groups were compared using the log-rank test.

Analyses were performed with IBM SPSS Statistics for Windows (version 20.0; Armonk, NY, IBM Corp), and statistical significance was considered when the “*p*” value was lower than 0.05 (two-tailed).

## Results

A total of 567 patients were included. Baseline characteristics are presented in Table 1. Of the total, 138 patients (24%) met the study criteria for NCA before the surgery date. Among these patients, the most frequent conditions were lower limb vascular disease, accounting for 72% of the cases, and stroke/TIA, accounting for 34%.

**Table 1:** Baseline characteristics prior to surgery.

Variables	Overall (567 patients)	NCA (138 patients)	No NCA (429 patients)	*p* value
Age (years)	67 (59–74)	70 (64–77)	66 (57–73)	<0.001
Male sex, *n* (%)	476 (84)	125 (90.6)	351 (81.8)	0.016
Weight (kg)	77 (69–85)	75 (68–81)	78 (69–86)	0.082
Height (cm)	169 (162–174)	168 (162–173)	170 (162–174)	0.577
Hypertension, *n* (%)	409 (72.4)	121 (87.7)	288 (67.4)	<0.001
Diabetes mellitus, *n* (%)	235 (41.4)	71 (51.4)	164 (38.2)	0.007
Dyslipidaemia, *n* (%)	389 (69)	111 (80)	278 (65)	0.001
Smoking, *n* (%)	363 (65)	102 (74)	261 (62)	0.013
COPD, *n* (%)	55 (9.7)	17 (12)	38 (8.0)	0.240
CKD, *n* (%)	16 (2.8)	11 (8.0)	5 (1.2)	<0.001
Liver failure, *n* (%)	9 (1.6)	3 (2.2)	6 (1.4)	0.550
Cancer diagnosis, *n* (%)	65 (12)	23 (17)	42 (10)	0.040
Atrial fibrillation, *n* (%)	46 (8)	13 (9,4)	33 (7,7)	0.590
Anticoagulation, *n* (%)	38 (6.7)	12 (8.8)	26 (6.1)	0.320
CHA_2_DS_2_VASC *n* (%)				
0–2	198 (52)	9 (10)	189 (65)	
3–5	168 (44)	68 (81)	100 (34)	0.001
6–7	11 (4)	7 (8)	4 (1)	
Neurological disorders, *n* (%)	7 (1.2)	6 (4.3)	1 (0.2)	<0.001
Lower limb vascular disease, *n* (%)	100 (17.6)	100 (72.5)	0 (0)	<0.001
Stroke/TIA, *n* (%)	47 (8.3)	47 (34.1)	0 (0)	<0.001
Carotid interventional treatment, *n* (%)	9 (1.6)	9 (6.5)	0 (0)	<0.001
Lower limb artery interventional treatment, *n* (%)	17 (3)	17 (12)	0 (0)	<0.001
Abdominal aortic aneurysm interventional treatment, *n* (%)	12 (2.1)	12 (8.7)	0 (0)	<0.001
Coronary stenting, *n* (%)	99 (17.5)	28 (20.0)	71 (16.6)	0.360
Prior CABG, *n* (%)	3 (0.5)	1 (0.7)	2 (0.5)	0.560
LVEF (%)	60 (45–60)	55 (42–60)	60 (45–60)	0.075
Estimated mortality (EuroSCORE I)	2.95 (1.7–5.7)	5.6 (3.2–9.2)	2.45 (1.5–4.5)	<0.001
Left main disease, *n* (%)	196 (34.8)	53 (39.0)	143 (33.5)	0.250
Proximal LAD disease, *n* (%)	344 (61)	76 (56)	268 (63)	0.180
Three vessel disease *n* (%)	420 (74.2)	113 (82.5)	307 (72.0)	0.010
eGFR (ml/min/1.73 m^2^)	85.8 (68.2–95.2)	77 (55.7–91.1)	87 (71.6–96.4)	<0.001
ACS as the cause of CABG, *n* (%)	230 (40.6)	43 (31.2)	187 (43.6)	0.010

Quantitative variables are expressed as medians and interquartile ranges.

ACS, acute coronary syndrome; CABG, coronary artery bypass graft; CKD, chronic kidney disease (glomerular filtration rate < 60 mL/min or previous diagnosis); COPD, chronic obstructive pulmonary disease; eGFR, estimated glomerular filtration rate; CKD-EPI, chronic kidney disease epidemiology collaboration equation; LVEF, left ventricular ejection fraction; NCA, non-coronary atherosclerosis; TIA, transient ischaemic attack.

Patients in the NCA group were older, predominantly male, and had a higher burden of traditional cardiovascular risk factors, as well as a high prevalence of kidney failure and oncological disease. In addition, patients with NCA had a higher EuroSCORE I than those without NCA. Of the total, 17.5% of patients had undergone a previous percutaneous revascularization procedure and three patients had undergone a previous CABG, with no significant differences between groups.

Moreover, acute coronary syndromes (ACS) accounted for around 41% of CABG procedures, being more frequent in the group without NCA. A total of 33% of patients had left main disease, and 66% had left anterior descendent artery (LAD) stenosis, with no differences between groups. Three-vessel disease was more frequent in the NCA group (82.5% vs. 72%).

Regarding technical issues of surgery, on-pump CABG was more frequently performed in the non-NCA group (82.2% vs. 71.7%). In approximately 98% of cases, a left internal mammary artery (LIMA) graft was used, with no differences between groups, whereas complete revascularization was more frequently achieved in non-NCA patients (76.5% vs. 68.0%) (Table 2).

**Table 2:** Surgical features and postoperative characteristics and complications.

Variables	Overall (567 patients)	NCA (138 patients)	No NCA (429 patients)	*p*-Value
On-pump surgery, *n* (%)	449 (79.6)	99 (71.7)	350 (82.2)	0.010
On-pump time, min	75 (45–104)	75 (0–107)	75 (48–102)	0.468
LIMA graft, *n* (%)	556 (98.1)	135 (97.8)	421 (98.1)	0.730
Complete revascularization, *n* (%)	422 (74.4)	94 (68.0)	328 (76.5)	0.050
Number of grafts, *n* (%)				
1	34 (6.0)	15 (11.0)	19 (4.5)	0.040
2	197 (34.7)	45 (32.6)	152 (35.0)	
≥3	335 (59)	78 (56)	257 (60)	
Arterial grafts, *n* (%)	236 (41.6)	55 (40.0)	181 (42.2)	0.690
LVEF (%)	55 (50–60)	55 (43–60)	55 (50–60)	0.010
AF, *n* (%)	49 (8.7)	8 (5.8)	41 (9.6)	0.220
ASA, *n* (%)	530 (94.3)	125 (92.6)	405 (94.8)	0.390
Antiplatelet other than ASA, *n* (%)	68 (12.1)	22 (16.3)	46 (11)	0.090
DAPT, *n* (%)	42 (7.5)	14 (10.4)	28 (6.6)	0.188
ACEI or ARB, *n* (%)	323 (57.5)	85 (63.0)	238 (56.0)	0.160
Beta-blockers, *n* (%)	501 (89)	119 (88.1)	382 (89.5)	0.630
Anticoagulation, *n* (%)	55 (9.8)	14 (10.4)	41 (9.6)	0.860
Statins, *n* (%)	554 (98.6)	131 (97.0)	423 (99.1)	0.090
AKI, *n* (%)	23 (4.1)	10 (7.3)	13 (3.1)	0.040
Infections, *n* (%)	49 (8.6)	10 (7.2)	39 (9.1)	0.600
HF, *n* (%)	47 (8.4)	14 (10.4)	33 (8.0)	0.360
AMI, *n* (%)	14 (2.5)	5 (3.7)	9 (2.1)	0.34
Stroke, *n* (%)	20 (3.5)	8 (5.8)	12 (2.8)	0.11
TIA, *n* (%)	3 (0.5)	0 (0.0)	3 (0.7)	1.00

Quantitative variables are expressed as medians and interquartile ranges.

ACEI, angiotensin-convertase enzyme inhibitors; AF, atrial fibrillation; AKI, acute kidney injury; AMI, acute myocardial infarction; ARB, angiotensin-receptor blockers; ASA, acetylsalicylic acid; DAPT, double antiplatelet therapy; HF, heart failure; LVEF, left ventricular ejection fraction; LIMA, left internal mammary artery; NCA, non-coronary atherosclerosis; TIA, transient ischaemic attack.

Complications before discharge are also detailed in Table 2. No differences were observed among groups, except for acute kidney injury (AKI), which occurred more frequently in the NCA group (7.3% vs. 3.1%).

There were no differences in medications at discharge nor in the rates of double antiplatelet therapy, which reached only 7% of patients.

Although the incidence of atrial fibrillation (AF) prior to surgery did not differ between groups, CHA_2_DS_2_VASc was significantly higher in the NCA group (Table 1). Moreover, there were no differences in the occurrence of AF after surgery. Seventy-two percent of patients who developed postoperative AF did not receive anticoagulation treatment on discharge, as arrhythmia was considered an immediate side effect of surgery. Chronic anticoagulation therapy at discharge was similar between both groups.

### Predictors of the primary outcome

After a median follow-up of 4.2 (2.3–6.9) years, 122 patients reached the primary outcome, with 73 patients (17%) in the non-NCA group compared to 49 patients (35.5%) in the NCA group (*p* < 0.001) (Table 3).

**Table 3:** Variables of the primary outcome.

Variables	NCA group 49 patients	Non-NCA group 73 patients	*p*-Value
TIA/stroke *n* (%)	8 (5.8)	15 (3.5)	0.11
AMI	5 (3.7)	9 (2.1)	0.34
New revascularization	7 (5.1)	18 (4.2)	0.63
Death	38 (27)	38 (9)	<0.001

AMI, acute myocardial infarction; NCA, non-coronary atherosclerosis; TIA, transient ischaemic attack.

The association of clinically relevant variables with the occurrence of the primary outcome in univariate Cox analysis is presented in Table 4.

**Table 4:** Univariate Cox regression analysis for the primary outcome.

Variables	*p*-Value	HR (CI 95%)
Age	<0.001	1.05 (1.03–1.07)
Sex	0.75	0.92 (0.56–1.51)
Diabetes mellitus	0.003	1.71 (1.20–2.45)
Hypertension	0.012	1.73 (1.10–2.73)
Dyslipidaemia	0.650	1.09 (0.74–1.60)
Smoking	0.490	1.14 (0.77–1.67)
Liver failure	0.070	2.94 (1.08–8.00)
COPD	0.770	1.09 (0.61–1.93)
Previous stenting	0.070	1.49 (0.97–2.29)
EuroSCORE I	0.008	1.02 (1.01–1.04)
LVEF prior to surgery	0.010	0.98 (0.96–0.99)
Cancer	0.170	1.45 (0.86–2.43)
Haemoglobin prior to surgery	<0.001	0.83 (0.75–0.92)
Albumin prior to surgery	<0.001	0.37 (0.25–0.54)
eGFR prior to surgery	<0.001	0.98 (0.97–0.98)
Acenocumarol prior to surgery	0.070	1.69 (0.98–2.91)
AF	0.100	1.57 (0.93–2.62)
Surgery indication	0.960	1.00 (0.70–1.44)
CAD	0.800	1.05 (0.69–1.60)
Left main disease	0.190	1.27 (0.88–1.82)
On-pump	0.007	0.56 (0.38–0.84)
LIMA use	0.020	0.32 (0.14–0.73)
Clamping time	0.010	0.99 (0.99–0.99)
Complete revascularization	0.390	0.84 (0.57–1.23)
Number of grafts	0.030	0.74 (0.56–0.97)
Arterial grafts	0.070	1.38 (0.97–1.98)
NCA	<0.001	2.44 (1.70–3.51)
ASA after surgery	0.960	0.99 (0.48–2.05)
Clopidogrel after surgery	0.880	1.04 (0.60–1.79)
Acenocumarol after surgery	0.080	1.58 (0.96–2.59)
Statins after surgery	0.66	0.76 (0.24–2.42)
Beta-blockers after surgery	0.09	0.63 (0.38–1.05)
ACEI/ARB post-surgery	0.18	1.28 (0.88–1.85)

ACEI, angiotensin-convertase enzyme inhibitor; ARB, angiotensin-receptor blocker; ASA, acetylsalicylic acid; CAD, coronary artery disease; COPD, chronic obstruction pulmonary disease; eGFR, estimated glomerular filtration rate by CKD-EPI (chronic kidney disease epidemiology collaboration equation); LVEF, left ventricular ejection fraction; LIMA, left internal mammary artery; NCA, non-coronary atherosclerosis.

**Figure 1:**
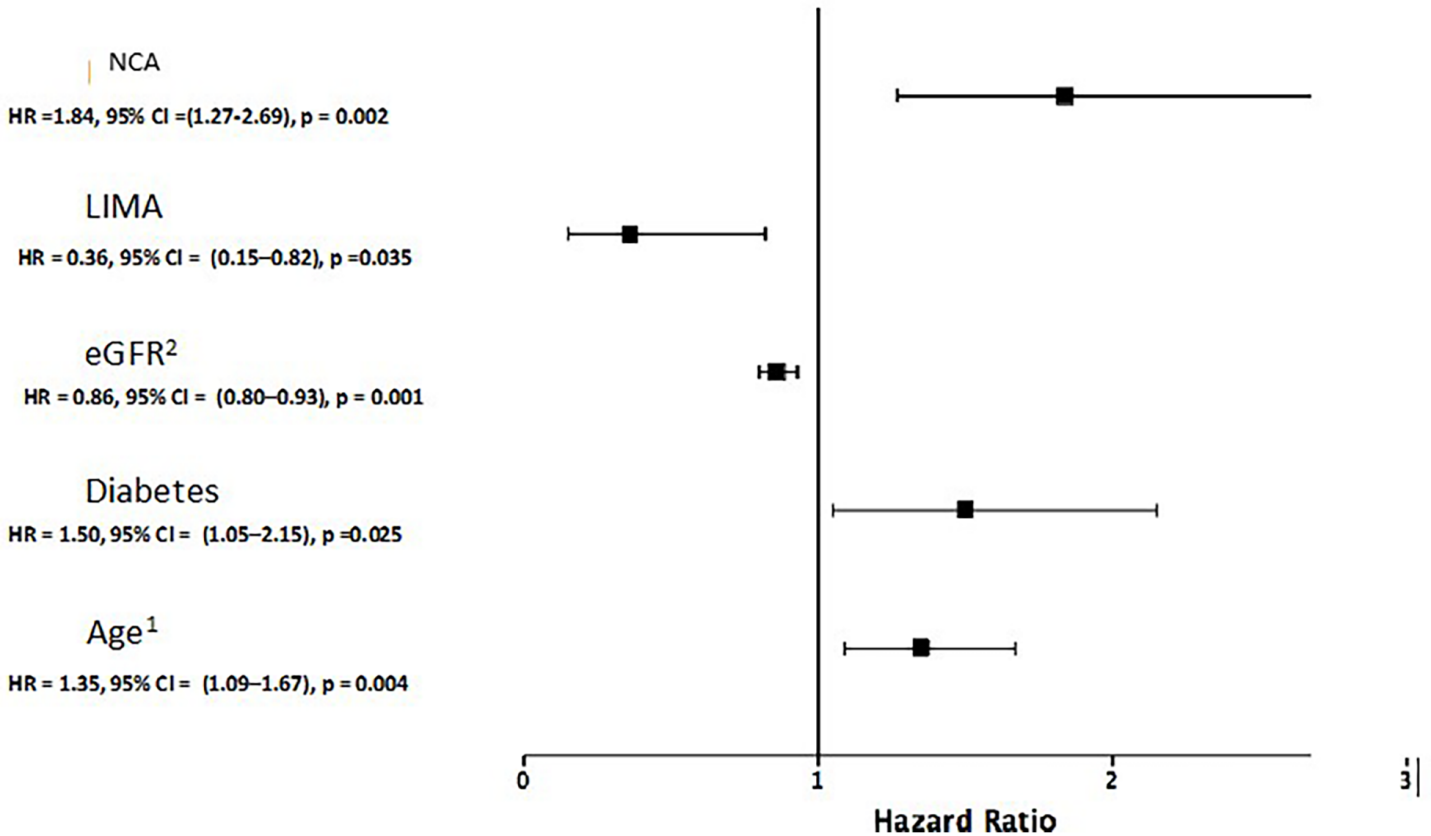
Final Cox regression multivariate model for the occurrence of the primary outcome. ^1^For each 10 years increase. ^2^For each 10 ml/min/1.73 m^2^ increase. eGFR: Estimated glomerular filtration rate by CKD-EPI (Chronic Kidney Disease Epidemiology Collaboration equation). LIMA, left internal mammary artery; NCA, non-coronary atherosclerosis.

In multivariate analysis, NCA, older age, diabetes mellitus, worse eGFR, and non-use of LIMA at surgery were independent predictors of the primary outcome (Figure 1).

**Figure 2:**
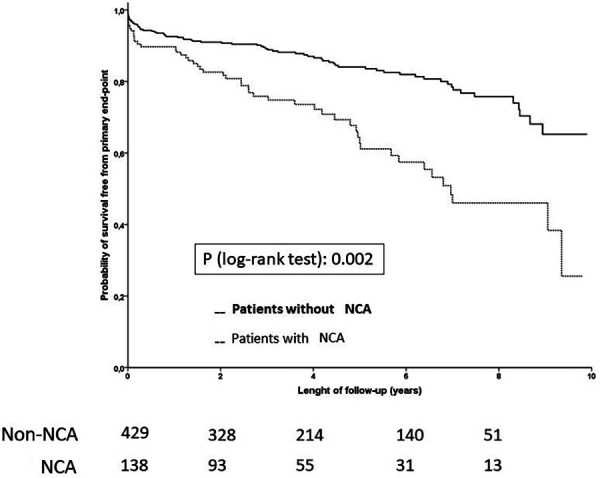
Kaplan-Meier curves for the primary outcome. NCA, non-coronary atherosclerosis.

Figure 2 shows the Kaplan–Meier curves for the occurrence of the primary outcome in both groups.

### Predictors of the secondary outcome

A total of 135 patients reached the secondary outcome, with 18.6% of patients in the non-NCA group (80 patients) compared to 40% of patients in the NCA group (55 patients). Lower extremity revascularization occurred in 11.4% of patients in the NCA group (16 patients) after follow-up compared to 0.9% of patients (4 patients) in the non-NCA group (*p* < 0.001), and AAA surgery was performed in 5 patients (3.7%) in the NCA group compared to 4 patients (0.9%) in the non-NCA group (*p* = 0.04).

The association of clinically relevant variables with the occurrence of the secondary outcome in univariate Cox analysis is detailed in the Supplemental Material.

In multivariate analysis, NCA and non-use of LIMA were independent predictors of the secondary outcome. Moreover, for each 10 mL/min worsening of the estimated glomerular filtration rate, the risk increased by 15% (Table 5).

**Table 5:** Multivariate analysis for the secondary outcome.

Variables	*p-*Value	HR (CI 95%)
eGFR^a^	<0.001	0.85 (0.79–0.91)
LIMA	0.020	0.34 (0.15–0.78)
NCA	<0.001	2.18 (1.53–3.11)

^a^ For each 10 mL/min/1.73 m^2^ increase.

eGFR, estimated glomerular filtration rate by CKD-EPI (chronic kidney disease epidemiology collaboration equation); LIMA, left internal mammary artery; NCA, non-coronary atherosclerosis.

**Figure 3:**
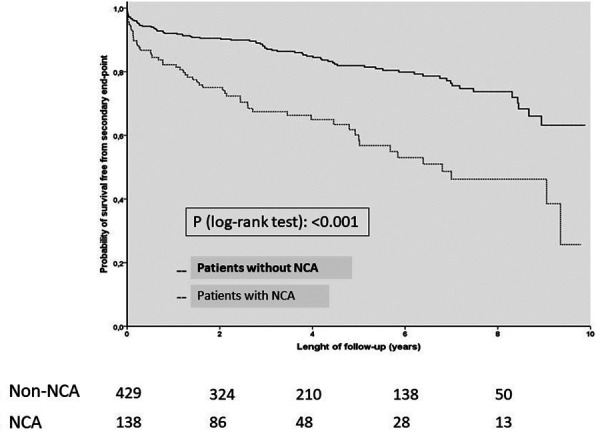
Kaplan-Meier curves for the secondary outcome. NCA, Non-coronary atherosclerosis.

Figure 3 shows the Kaplan–Meier curves for the occurrence of the secondary outcome in both groups.

### Mortality

The median follow-up was 3.6 years (IQR 1.9–6.4) in the NCA group and 4.4 years (IQR 2.5–7.0) in the non-PAD group. Mortality was higher in the NCA group (27% vs. 9%, *p* < 0.001) (Table 6). On the other hand, mortality within the first 30 days after surgery was similar in both groups (2.9% in the NCA group and 1.2% in the non-NCA group, *p *= 0.23).

**Table 6:** Causes of death.

	Total, *n* = 76 (13%), *n* (%)	NCA, *n* = 38 (27%), *n* (%)	Non-NCA, *n* = 38 (9%), *n* (%)	*p*-Value
Cardiac	16 (21)	10 (26.3)	6 (15.8)	0.235
Vascular	4 (5.3)	3 (7.9)	1 (2.8)	0.358
Neurologic	5 (6.6)	3 (7.9)	2 (5.3)	0.675
Infections	18 (23.7)	9 (23.7)	9 (23.7)	0.842
Cancer	15 (19.7)	7 (18.4)	8 (21.1)	0.817
Other	18 (23.7)	6 (15.8)	12 (31)	0.118

PAD, peripheral artery disease.

## Discussion

The main finding of our study was the confirmation that NCA is a strong predictor of worse prognosis in CAD patients undergoing CABG as a revascularization therapy. Thus, its presence increases the risk of TIA/stroke, AMI, new revascularization procedure, or death by 84% compared to patients without NCA.

In our study, NCA, older age, and diabetes mellitus were independent predictors of the primary outcome, while estimated glomerular filtration rate and use of LIMA were inversely associated with this outcome. Secondary outcome added the need for lower extremity revascularization or AAA surgery, with NCA once again emerging as a clearly determining factor, alongside impaired kidney function.

Traditional cardiovascular risk factors (smoking, hypertension, diabetes mellitus, and dyslipidaemia) and the ageing of the population are recognized as important determinants of NCA in all countries (13–15).

The BARI study previously demonstrated that searching for NCA, even when clinically silent, in CAD patients could be crucial for early detection of underlying conditions, and it could lead to more aggressive cardiovascular preventive treatment strategies (12, 16, 17).

When comparing our results with those from other studies, caution must be exercised due to the heterogeneous definitions of NCA. Some studies refer to PAD when diagnosing lower limb arterial disease (13, 18), while others include the existence of AAA and its repair treatments in the definition (12, 19, 20). Moreover, there have been studies (8, 21) that considered previous stroke/TIA as a form of PAD. As we aimed to include stroke and TIA as forms of previous peripheral atherosclerosis, we used the term NCA, including stroke and TIA on the one hand, along with the classical concept of PAD on the other hand, defined as LEAD, carotid disease, previous lower limb vascular surgery, or AAA.

The prevalence of NCA among our population was 24.3%, which was similar to other studies (17% in Nakamura et al., 26% in Birkmeyer et al., and 21% in Harskamp et al.). All these series described a similar incidence of older age, impaired renal function, and a higher frequency of cardiovascular risk factors in the PAD group (6, 8, 18, 21) compared with non-PAD patients.

Complications after CABG surgery were greater in previous studies in the PAD group (Harskamp et al.; Bonacchi et al.), with kidney failure up to 8.9% and a stroke rate of 2.7% (20, 21). In our study, the proportion of acute kidney injury after surgery was also higher in patients with NCA (7.3% vs. 3.1%), but there were no differences in terms of stroke. On the other hand, Chu et al. found no differences in the complication rate in the NCA group (13).

Considering mortality, the group of NCA patients may represent a subset of patients with a more aggressive form of CAD that progresses much faster, thus contributing to the increased late mortality observed in the NCA patients. It is important to differentiate between early (within 30 days after surgery) and long-term mortality. Birkmeyer et al. found a 2.4 times higher in-hospital mortality in patients with PAD (8). Our results, just like those of Harskamp et al. (21), Nakamura et al. (18), or van Straten et al. (19), did not show any significant difference in early mortality. However, in the long term, better survival was noted among the participants in the non-NCA group (survival was 90% vs. 72%).

Concerning causes of death, cardiovascular disease accounted for 34% in the NCA group vs. 18% in the non-NCA group, although it did not reach statistical significance. Birkmeyer et al. showed an excess of deaths due to heart failure and arrhythmia in the NCA group (8). Meanwhile, the BARI study highlighted that the main cause of mortality among PAD patients was cardiac, although the percentages of deaths from other causes were also more frequent (16).

Thus, with this body of evidence, it seems reasonable to recommend strategies to assess for the presence of NCA among patients with established CAD or in patients with high cardiovascular risk features. These findings suggest that NCA continues to identify a subgroup at particularly high risk for adverse cardiovascular outcomes that might benefit from more intensive secondary prevention. However, up to date, the single study that addressed this topic has failed to reach significance, although it had important limitations, such as the inclusion of high-risk patients in both arms (5).

On the other hand, PAD has been described as a treatment modifier in terms of the duration of dual antiplatelet therapy in patients undergoing percutaneous coronary intervention, with a prolonged regimen (up to 24 months) associated with a significantly lower risk of death and atherothrombotic events and unaffected risk of bleeding (22). Nevertheless, although clinical practice guidelines still recommend double antiplatelet therapy in ACS (9), irrespective of the revascularization strategy, there have been contradictory data about it among studies. This could be due to heterogeneous designs related to on-pump use and indication of surgery, among other factors (23). In our study, we did not find significant differences, probably due to the small sample size of patients discharged with double antiplatelet therapy.

### Limitations

This was a retrospective observational analysis, and thus, patients with subclinical NCA were not identified. The inclusion and exclusion criteria of this study may limit the possibility of extending our findings to other populations. The type of major adverse limb events was not available, so we could only include vascular limb intervention. Finally, the limited sample size precluded us from obtaining information about other interesting aspects, such as potential interactions between NCA and the use of dual antiplatelet therapy.

## Conclusions

Among patients treated with CABG surgery, the presence of NCA confers a higher risk of death and doubles the incidence of the combined outcome of TIA/stroke, AMI, new revascularization therapy, lower limb or AAA surgery, and death.

## Data Availability

The original contributions presented in the study are included in the article/Supplementary Material; further inquiries can be directed to the corresponding author.
