# Supplementary material for: Non-coronary atherosclerosis: a marker of poor prognosis in patients undergoing coronary artery bypass surgery
Source: Front Cardiovasc Med. 2024 Feb 23;11:1305162. doi: 10.3389/fcvm.2024.1305162 (PMC10921089; doi:10.3389/fcvm.2024.1305162)
Supplement: Supplementary file 1 [file Table1.docx]

**SUPPLEMENTAL MATERIAL:**

Univariate analysis for the secondary outcome

| Variables | P value | HR (CI 95%) |
| --- | --- | --- |
| Age | <0.001 | 1.04 (1.02-1.06) |
| Sex | 0.550 | 0.86 (0.54-1.39) |
| Diabetes Mellitus | 0.002 | 1.70 (1.21-2.39) |
| Hypertension | 0.005 | 1.79 (1.16-2.76) |
| Dyslipidaemia | 0.810 | 1.04 (0.72-1.50) |
| Smoking | 0.220 | 1.25 (0.86-1.81) |
| Hepatopathy | 0.080 | 2.73 (1.01-7.41) |
| COPD | 0.870 | 1.04 (0.60-1.82) |
| Previous stenting | 0.180 | 1.33 (0.87-2.02) |
| Euroscore I | 0.007 | 1.02 (1.01-1.04) |
| LVEF prior surgery | 0.007 | 0.98 (0.96-0.99) |
| Cancer | 0.040 | 1.64 (1.03-2.62) |
| Haemoglobin prior surgery | <0.001 | 0.82 (0.74-0.90) |
| Albumin prior surgery | <0.001 | 0.45 (0.31-0.65) |
| eGFR prior surgery | <0.001 | 0.82 (0.77-0.88) |
| Acenocumarol prior surgery | 0.070 | 1.67 (0.99-2.83) |
| AF | 0.170 | 1.44 (0.86-2.40) |
| Surgery causes | 0.600 | 1.09 (0.77-1.55) |
| CAD | 0.280 | 1.24 (0.82-1.87) |
| Left main disease | 0.470 | 1.13 (0.80-1.61) |
| On-pump use | 0.003 | 0.55 (0.38-0.81) |
| LIMA use | 0.030 | 0.35 (0.15-0.81) |
| Clamping time | 0.010 | 0.99 (0.99-0.99) |
| Complete revascularization | 0.430 | 0.86 (0.59-1.24) |
| Number of grafts | 0.060 | 0.77 (0.59-1.00) |
| Arterial grafts | 0.030 | 1.43 (1.02-2.00) |
| NCA | <0.001 | 2.58 (1.83-3.65) |
| ASA post-surgery | 0.890 | 0.95 (0.48-1.87) |
| Clopidogrel post-surgery | 0.970 | 1.00 (0.59-1.70) |
| Acenocumarol post-surgery | 0.170 | 1.42 (0.87-2.31) |
| Statins post-surgery | 0.770 | 0.84 (0.26-2.67) |
| Beta-blockers post-surgery | 0.080 | 0.64 (0.40-1.04) |
| ACEI/ARA II post-surgery | 0.040 | 1.43 (1.00-2.05) |

ACEI: angiotensin-convertase enzyme inhibitors. ARB: Angiotensin-receptor blockers. ASA: Acetylsalicylic Acid. CAD: coronary artery disease. COPD: chronic obstruction pulmonary disease. eGFR: estimated glomerular filtration rate by CKD-EPI (Chronic Kidney Disease Epidemiology Collaboration equation). LVEF: left ventricular ejection fraction. LIMA: left internal mammary artery. NCA: non-coronary atherosclerosis.

| ***Variable*** | ***Definition*** |
| --- | --- |
| **AAA** | Vascular diagnosis or previous treatment of AAA. Abdominal aorta diameter >3 cm. |
| **Stroke** | Diagnosis by Neurology, suggestive image in cerebral CT/RM. |
| **TIA** | Diagnosis by Neurology, <24h length, no sequels, normal cerebral CT/RM. |
| **Unstable angina** | Resting angina that requires intravenous nitrates until the arrival in the operating room. |
| **Carotid artery disease** | Previous treatment (previous stent/endarterectomy) or Doppler ultrasound stenosis >50%. |
| **Peripheral arterial disease (PAD)** | One or more of the following:  - Lower limbs claudication  - Carotid occlusion or stenosis > 50 %.  - Previous vascular surgery in abdominal aorta carotid or lower limbs. |
| **Lower limb arterial disease** | Diagnosis by Vascular surgeon or previous endovascular treatment or by surgery (íleo-femoro-poplíteo). |
| **Cancer** | Diagnosis de neoplasm in any location. |
| **CHA2DS2VASC** | \| ***Risk factor*** \| ***Punctuation*** \| \| --- \| --- \| \| *Heart failure* \| 1 \| \| *HBP* \| 1 \| \| *Age ≥ 75 years* \| 2 \| \| *Diabetes Mellitus* \| 1 \| \| *Stroke, TIA or previous thromboembolism* \| 2 \| \| *Vascular disease* \| 1 \| \| *Age 65-74 years* \| 1 \| \| *Female* \| 1 \| |
| **AAA surgery** | Abdominal aortic aneurysm surgery |
| **Emergent cardiac surgery** | Surgery performed before the next day of work |
| **Neurological disorders** | Neurological damage that severely affect to everyday life. |
| **Diabetes Mellitus** | Previous diagnosis and hypoglycemic treatment |
| **Dislipydaemia** | Previous diagnosis and hypolipidemia treatment |
| **Infective endocarditis** | In antibiotic treatment for endocarditis at the time of surgery |
| **Significant CAD** | Coronary obstruction >70%; for left main coronary >50%. |
| **COPD** | Previous diagnosis, that requires prolonged treatment with bronchodilators or steroids. |
| **EuroScore I** | \| ***Risk factor*** \| ***value*** \| \| --- \| --- \| \| *Age (years)*  60-64  65-69  70-74  75-79  80-84  85-89  90-94  95-100 \| 1  2  3  4  5  6  7  8 \| \| *Female* \| 1 \| \| *LVEF (%)*  <30  30-50 \| 3  1 \| \| *Renal dysfunction* \| 2 \| \| *Lower limbs disease* \| 2 \| \| *Respiratory disease* \| 1 \| \| *Cerebrovascular disease* \| 2 \| \| *Previous cardiac surgery* \| 3 \| \| *Active endocarditis* \| 3 \| \| *Critical preoperative situation* \| 3 \| \| *Unstable angina* \| 2 \| \| *Recent myocardial infartion* \| 2 \| \| *Pulmonary hypertension* \| 2 \| \| *Emergent surgery* \| 2 \| \| *Surgery other tan isolated coronary surgery* \| 2 \| \| *Ascending Aorta surgery* \| 3 \| \| *Rupture of the interventricular septum* \| 4 \| |
| **AF prior surgery** | Previous diagnosis. Use of antarrhythmic or anticoagulant treatment. |
| **AF post-surgery** | Episodes lasting more than an hour or that require treatment to reverse it. |
| **LVEF post-surgery** | Left ventricular eyection fraction after coronary surgery at discharge. |
| **LVEF prior surgery** | Left ventricular eyection fraction prior to surgery. |
| **eGFR** | Calculated with the formula CKD-EPI |
| **Hepatopathy** | Cirrhosis or liver transplant |
| **High blood pressure (HBP)** | Previous diagnosis and with medical treatment |
| **Infection post-surgery** | Fever with increased reactants of acute phase and or need for antibitic or antifungal for suspected respiratory, urinary or any type of infection considered by the responsible doctor. |
| **AKI post-surgery** | Acute kidney injury after surgery: need for renal substitution or deterioration >50% creatinine regarding the baseline. |
| **CKD prior surgery** | Renal disease previous surgery |
| **Critical preoperative condition** | One or more of the following:  - Ventricular tachycardia/ ventricular fibrilation or recovered sudden death  - Preoperative heart massage  - Mechanical ventilation prior to anesthesia  - Need preoperative inotropics  - Intraaortic ball preoperative  - Preoperative acute renal failure (diuresis < 10 ml./h). |
| **Acenocumarol prior surgery** | Use of anticoagulants prior surgery (acenocumarol, direct-acting anticoagulants or heparina). |
| **Acenocumarol post-surgery** | Use of anticoagulants prior surgery (acenocumarol, direct-acting anticoagulants or heparina). 24 h after the surgery |
